# Liver X receptor activation promotes differentiation of regulatory T cells

**DOI:** 10.1371/journal.pone.0184985

**Published:** 2017-09-19

**Authors:** Martin Herold, Johanna Breuer, Stephanie Hucke, Percy Knolle, Nicholas Schwab, Heinz Wiendl, Luisa Klotz

**Affiliations:** 1 Department of Neurology, University of Muenster, Albert-Schweitzer-Campus 1, Muenster, Germany; 2 Institute of Molecular Immunology & Experimental Oncology, Technical University Munich, Munich, Germany; Universite Paris-Sud, FRANCE

## Abstract

The nuclear receptor Liver X Receptor (LXR) is a ligand-activated transcription factor that has been implicated in control of chronic inflammation by downregulating pro-inflammatory T cell responses. An impaired function of regulatory T cells, a subset of CD4+ T cells with a crucial role in maintaining lymphocytes homeostasis and immune regulation, is frequently observed in chronic inflammatory diseases. We observed that pharmacological activation of LXR in T cells not only resulted in a thorough suppression of Th1 and Th17 polarization in vitro, but also significantly induced regulatory T cells (Treg) cell differentiation in a receptor-specific fashion. In line with this, systemic LXR activation by oral treatment of mice with the LXR agonist GW3965 induced gut-associated regulatory T cells *in vivo*. Importantly, such LXR-activated Tregs had a higher suppressive capacity in functional *in vitro* coculture assays with effector T cells. Our data thus point towards a dual role of LXR-mediated control of inflammation by suppression of pro-inflammatory T cells and reciprocal induction of regulatory T cells.

## Introduction

Regulatory T cells (Treg) are a subset of CD4+ T cells that play a key role in prevention of autoimmune diseases, maintainance of immune homeostasis and modulation of immune responses during infection [1]. Forkhead box P3 (FoxP3), the master transcription factor of Treg, is crucial for development of suppressive function [2, 3].

Interestingly in a variety of autoimmune diseases, including both organ-specific (multiple sclerosis, type 1 diabetes) and systemic (rheumatoid arthritis) diseases a loss of Treg functionality has been observed, while Treg numbers were unaffected or even increased [4–6].

*In vivo*, Treg are comprised of either thymus-derived Treg (tTreg, also natural Treg (nTreg)) or differentiate from peripheral naïve CD4+ T cells (induced Treg–iTreg) [7]. Besides expressing FoxP3, Treg are characterized by constitutive CD25 expression and low / absent CD127 expression [8–11]. CD25 is part of a high-affinity IL-2 receptor (IL-2R) and is essential for generation, expansion and suppressive capacity of Treg [12, 13]. FoxP3 induces high expression of CD25 as well as expression of CTLA-4, a Treg-associated surface molecule [14]. CTLA-4 is also implicated in the suppressive capacity of Treg and mediates cell contact-dependent downregulation of the costimulatory molecules CD80 and CD86 on APCs [10, 14, 15], which results in tolerogenized dendritic cells (DCs) that further augment Treg induction [16, 17].

Induced Treg develop extrathymically from conventional CD4+ cells under inflammatory and non-inflammatory conditions [18]. Similar to nTreg, iTreg express FoxP3, CD25, and CTLA-4, and exhibit a potent suppressive capacity as their main feature [19]. Recently Helios, a member of the Ikaros transcription factor family, was identified as a potential marker to discriminate between nTreg and iTreg, as Helios is upregulated in nTreg compared to iTreg [20]. Although there is evidence, that Helios might be induced during T cell activation and proliferation in both subsets, Helios remains the best marker so far to distinguish nTreg from iTreg and allows discrimination in both humans and mice [21–24].

The liver X receptor (LXR) is a ligand-activated transcription factor that belongs to the group of nuclear receptors (NR) and exists in two isoforms: The first is LXRα, which is expressed specifically in liver, intestine, adipose tissue, lung and macrophages. The second is LXRβ, which is ubiquitously expressed [25–27]. Both isoforms are expressed by CD4+ T cells and macrophages [28, 29]. Ligand-based activation of LXRs leads to the formation of a heterodimer with retinoid X receptor (RXR), which in turn allows the regulation of genes with a central role in the modulation of cholesterol homeostasis and fatty acid metabolism [30]. Besides physiological ligands, such as oxysterols and intermediates of the biosynthetic cholesterol pathway, potent synthetic ligands are available including T0901317 and GW3965 [31].

Pharmacological LXR activation has been shown to be efficient in preclinical models of inflammation including atherosclerosis [32], contact dermatitis [33], rheumatoid arthritis [34], multiple sclerosis [35] and colitis [36]. LXR-deficient mice show a higher susceptibility and aggravated disease progression in the colitis model, which is linked to increased pro-inflammatory cytokine and chemokine expression [36]. In turn, pharmacological receptor activation ameliorated disease progression and increased survival. In a mouse model of multiple sclerosis pharmacological activation of LXR resulted in an ameliorative effect linked to decreased effector T cell responses and inhibition of IL-23 receptor and IL-17 expression [35, 37, 38]. Furthermore mice deficient for LXR were reported to develop an aggravated disease.

Taken together, these studies suggest that LXR is a strong negative regulator of pro-inflammatory processes [34–36] with a direct relevance in pro-inflammatory T cells. Interestingly, it is known that LXR-related receptor RXR regulates pro-inflammatory T cell differentiation while reciprocally inducing Treg differentiation when heterodimerizing with the retinoid acid receptor (RAR). Moreover, dietary changes in cholesterol uptake of chronic hepatitis C patients, known for increased levels of Th17 cells, were reported to result in increased expression of LXR and LXR-target genes while improving the Treg/Th17 balance in peripheral immune cells. This raised the question whether pharmacological LXR activation in T cells might also promote formation of functional Treg, thereby further supporting control of inflammation. Hence, we addressed the role of LXR-activation in Treg.

## Results

### LXR ligand GW3965 controls pro-inflammatory T cell polarization while reciprocally enhancing regulatory T cell differentiation

**Fig 1:**
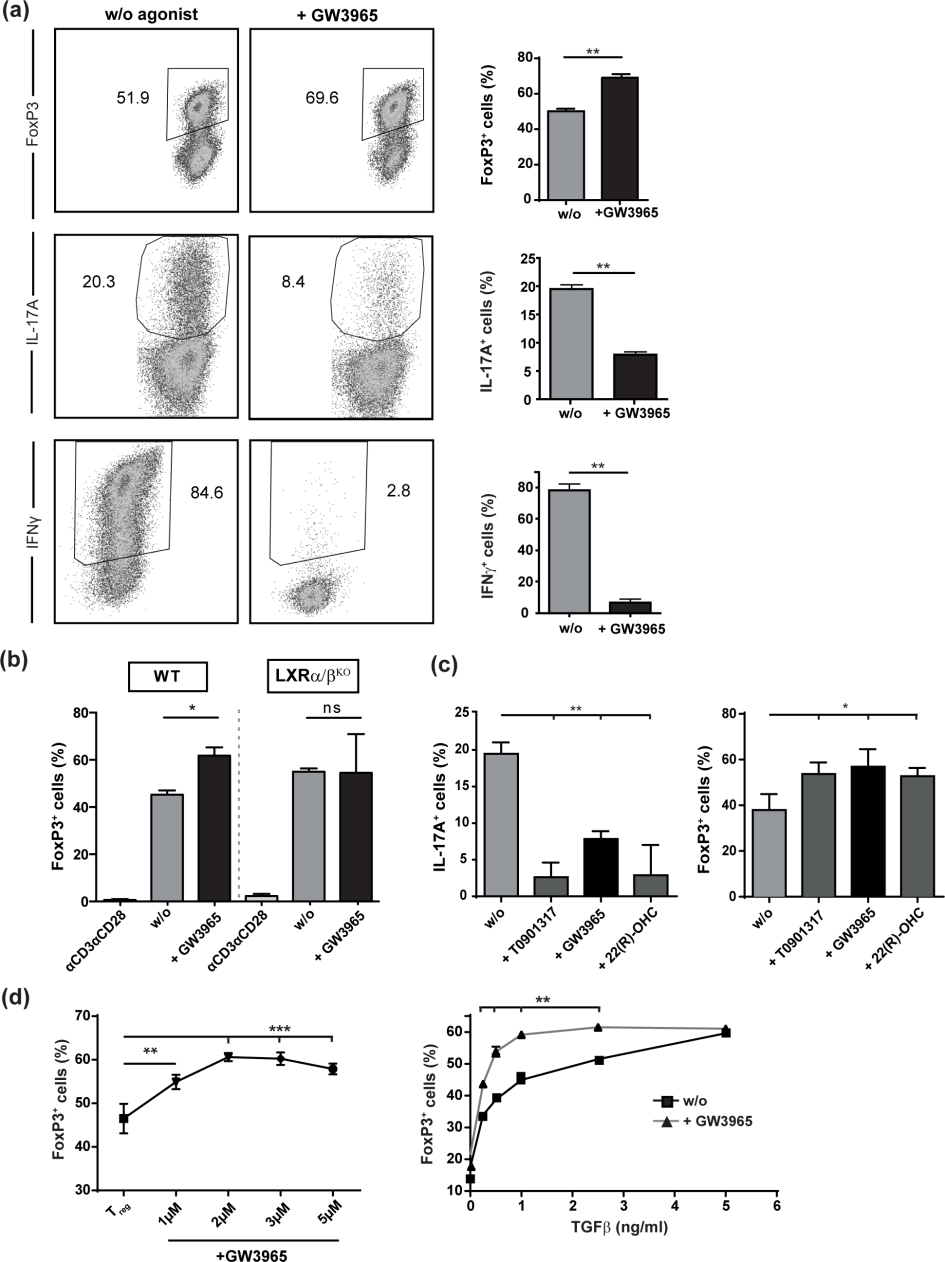
Pharmaceutical LXR activation controls pro-inflammatory Th1 and Th17 polarization while reciprocally enhancing regulatory T cell differentiation. (a-d) Purified CD4+ T cells from WT mice were subjected to *in vitro* Th17-, Th1- and Treg-differentiation in the absence (w/o) or presence of LXR agonists GW3965 (3μM), T0901317 (2μM) or 22(R)-OHC (20μM) for 72 hours and subsequently stained intracellularly for IL-17A, IFNγ, and FoxP3 expression. The percentage of positive cells was determined by flow cytometry with triplicates measured in each experiment. Data shows pooled results of three individual experiments. Graphs show percentage ± SEM. * p<0.05 **p<0.01 ***p<0.001.

To assess whether T cell differentiation is modulated by LXR activation, we *in vitro* differentiated CD4^+^ T cells with polarizing cytokines in the presence of TCR activation into either regulatory T cells (Treg) or pro-inflammatory Th1 and Th17 cells. In the presence of LXR activation using the pharmacological ligand GW3965, we observed significantly enhanced differentiation into FoxP3^+^ Tregs (Fig 1A), whereas, as expected from previously published data, Th1 and Th17 polarization was substantially suppressed [35]. Of note, the secretion of both IFNγ and IL-17A was significantly reduced under Treg polarizing conditions (S1A Fig), while IL-10, IL-4, GM-CSF and IL-6 secretion remain unaffected (S1B Fig). We next sought to clarify whether GW3965-dependent promotion of Treg differentiation is specific for LXR. To this end, LXRα/β^KO^ T cells where exposed to Treg-polarizing conditions in the presence of LXR agonist GW3965. Importantly, enhanced Treg differentiation was completely abrogated in LXRα/β-deficient T cells (Fig 1B), demonstrating that effects of GW3965 were indeed receptor-specific. Furthermore, we addressed the capacity of other commonly used LXR agonists, i.e. synthetic ligand T0901317 and endogenous LXR ligand 22(R)-OHC, to modulate T cell differentiation. Both ligands elicited a comparable suppressive effect on Th17 cells (Fig 1C, *left*) and inductive effect on Treg (Fig 1C, *right*). Moreover, GW3965-based LXR activation acted in a dose-dependent manner in enhancement of Treg differentiation (Fig 1D, *left*). Interestingly, LXR-mediated Treg induction was most prominent in the presence of lower TGFβ concentrations (Fig 1D, *right*) suggesting that LXR might promote Treg induction especially under suboptimal Treg conditions.

Taken together, LXR activation not only restricts pro-inflammatory T cell generation, i.e. Th17 and Th1, but also reciprocally enhances Treg differentiation.

### LXR-activation in vivo induces gut-associated regulatory T cells with enhanced suppressive capacity

**Fig 2:**
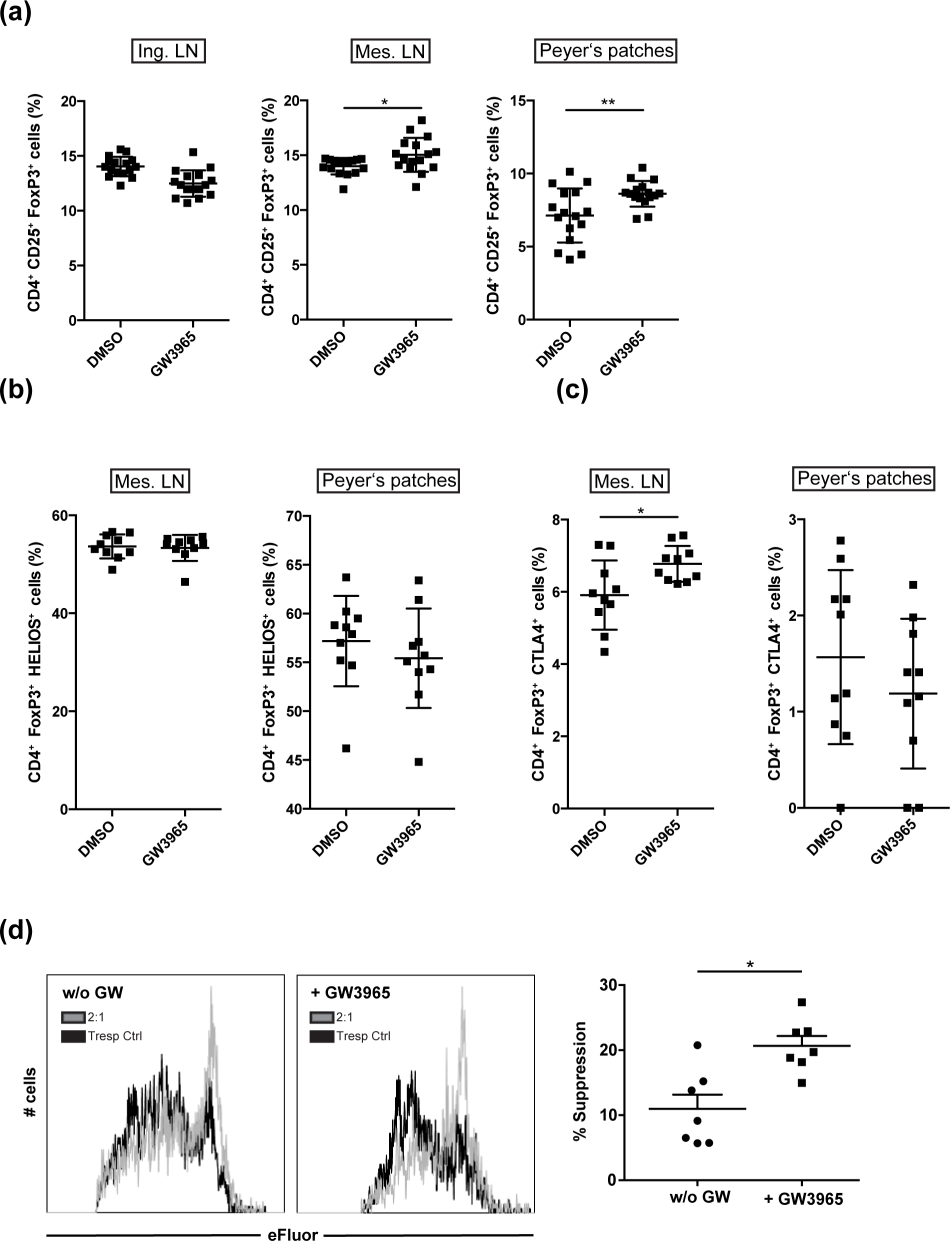
LXR-activation induces gut-associated regulatory T cells *in vivo* with enhanced suppressive capacity. (a) FoxP3 expression of CD4^+^ T cells was assessed by flow cytometry. T cells were isolated from inguinal LN (Ing. LN), mesenteric LN (Mes. LN) and peyer’s patches (PP) of wildtype mice treated orally for seven days with DMSO (vehicle) or GW3965 (n = 16). Treg population (CD4+, FoxP3+) from the gut-associated tissue (mes. LN, PP) was further analyzed for expression of (b) Helios and (c) CTLA-4 (n = 10). (d) Murine splenic Treg, which were incubated with DMSO or GW3965, were functionally characterized in a suppression assay. Suppression assays were performed by coculturing Treg (Treg; CD4+CD25+) with allogenic responder T cells (Tresp; CD4+CD25-) in a 2:1 ratio in the presence of anti-CD3/CD28 beads (cell to bead ratio = 30:1) and GW3965 (1.5 μM) or vehicle control (DMSO), respectively. Proliferation was assessed by flow cytometry (n = 7) and suppression was calculated and is displayed as % suppression (as described in chapter 4.6). Graphs show percentage ± SEM. *p<0.05 **p<0.01.

We next asked whether LXR activation induces Treg also *in vivo*, which could potentially help to regulation of enhanced effector T cell responses. *In vivo*, Treg play a key role in the maintenance of gut homeostasis as both, nTreg and iTreg subpopulations, contribute to colitis suppression [20]. We therefore aimed to assess the impact of systemic LXR activation on gut-associated Treg. To this end, we treated wildtype mice with GW3965 by daily oral gavage and analyzed the frequency of Treg after seven days of treatment in gut-associated mesenteric lymph nodes (Mes. LN) as well as gut-associated lymphoid structures, i.e. Peyer’s Patches and non-draining inguinal LN (Ing. LN). We observed a significant increase in the frequency of CD4^+^CD25^+^FoxP3^+^ Treg in both, mesenteric LN (Mes. LN) as well as in Payer’s patches (Fig 2A) upon LXR activation, whereas no alteration of Treg frequency was observed in inguinal LN (Fig 2A, Ing. LN). Flow-cytometric analysis of Helios expression in Treg of mesenteric LN and Peyer’s Patches revealed no differences in Helios expression, indicating that LXR activation does not promote recruitment of thymic Treg to gut-associated lymphoid tissue, but instead results in induction of naïve CD4^+^ T cells to differentiate into Treg (Fig 2B). Furthermore, it was of interest to evaluate the potential suppressive character of LXR-induced gut-associated Treg. We therefore investigated CTLA-4 expression on Treg in mesenteric LN and Peyer’s Patches (Fig 2C) upon daily oral treatment of mice with the LXR ligand GW3965. Interestingly, we observed an increase in CTLA-4 expression on Treg in mesenteric LN, whereas no alteration in CTLA-4 expression was observed on Peyer’s Patches-derived Treg (Fig 2C). This suggests that, at least in mesenteric LN, Treg not only increase in frequency but also in their suppressive function. Taken together, also *in vivo* LXR activation results in induction of Treg, which is associated with an increase in CTLA-4 expression.

We next wanted to assess whether LXR activation additionally modulates the suppressive capacity of Treg. Accordingly, we analyzed whether LXR-induced Treg display an increased capacity to restrict proliferation of activated CD4^+^ T cells and made use of a classical *in vitro* suppression assay setup. Here, isolated splenic Treg were treated with GW3965 for LXR activation for 24h or were left untreated, before setup of a coculture with effector T cells in the presence or absence of the agonist. We observed that LXR-activated Treg exhibited significantly enhanced suppressive properties when compared to untreated control Treg (Fig 2D) and induced a significant reduction of IFNγ and IL-17A secretion (S1C Fig), while IL-10, IL-4, GM-CSF and IL-6 secretion remain unaffected (S1D Fig.). This demonstrates that LXR-activation not only results in an increase in Treg numbers, but more importantly functionally alters Treg, which then display enhanced suppressive capacity.

## Discussion

In the current study, we investigated the influence of pharmacological LXR activation on the polarization of CD4+ T cells. We here report that LXR suppresses pro-inflammatory T cell differentiation while reciprocally promoting Treg differentiation *in vitro* and inducing gut-associated Treg *in vivo*. Furthermore, LXR-activated Treg not only increase in numbers but also exhibit enhanced suppressive capacity. Of interest, LXR activation was especially effective in Treg induction under suboptimal TGFβ concentrations.

Irrespective of their subtype, inflammatory effector T cells are controlled by regulatory immune cells, such as Treg. The pivotal role of Treg has been revealed in various animal models for inflammatory diseases [31–33] and mutations in the FoxP3 gene lead to the development of the fatal autoimmune disorder immunodysregulation polyendocrinopathy enteropathy X-linked (IPEX) syndrome (or scurfy phenotype in mice), that results in systemic autoimmunity [39–41]. Also, the conditional depletion of Treg in adult mice results in the development of autoimmunity [42], further emphasizing their importance in immune homeostasis.

NRs have been shown to be involved in the homeostasis of effector T cell and Treg balance, and genetic deletion of NRs often results in dysregulated immune responses. For example, deletion of either PPARγ [43], PPARδ [44], or LXRα/β [35] results in aggravated disease progression in experimental autoimmune encephalomyelitis (EAE), the animal model of Multiple Sclerosis. Aggravated disease is characterized by enhanced frequencies of pro-inflammatory T cell subsets, whereas ligand-mediated activation ameliorates clinical signs and restricts Th1 and Th17 effector responses.

Interestingly, ligand-based activation of the NR aryl hydrocarbon receptor (AHR) induces Treg that suppress CNS autoimmunity in EAE by a TGF-β1-dependent mechanism [45]. Furthermore, RXR activation reciprocally induces Treg and suppresses Th17 differentiation [46] and transferred RAR-activated Treg are more potent suppressors in an acute, small intestinal inflammation model compared with control Treg [47]. In spite of the apparent association between LXR and RXR, so far only suppressive effects on pro-inflammatory T cells have been reported for LXR. We here observed strong induction of Treg differentiation upon pharmacological LXR activation *in vitro*, while reciprocally Th1 and Th17 differentiation was suppressed. LXR-mediated Treg induction was most prominent under suboptimal TGFβ concentrations, which indicates that this mechanism might be especially relevant under chronic inflammatory conditions with potentially disturbed Treg formation [48, 49].

Importantly, we could show that pharmacological LXR activation *in vivo* resulted in a clear induction of Treg in gut-associated lymphoid tissue, i.e. Peyer’s patches and mesenteric LN, which was not observed in unrelated lymphatic tissue. Moreover, the increase in Treg frequencies was not due to recruitment of thymic-derived Treg, as the frequency of Helios positive Treg remained unchanged under GW3965-treatment [24]. These findings indicate that LXR activation via oral application of a LXR agonist might be especially relevant for control of intestinal autoimmune responses, such as in colitis. However, in light of the increasingly acknowledged role of intestinal immune responses for control of systemic autoimmunity, this effect might also be relevant in other autoimmune diseases such as Multiple sclerosis, arthritis or type 1 diabetes [50–53]. Furthermore, CTLA-4 expression was induced on Treg in mice orally treated with GW3965. This allows the hypothesis that, besides locally increased differentiation of Treg, LXR activation also enhances the suppressive capacity of gut-associated Treg. This hypothesis is supported by our finding that LXR-activation enhances the suppressive capacity of splenic Treg *in vitro*. After an exposition time of 96h, LXR activation was capable to boost the Treg-mediated suppression of effector T cells, thus indicating that LXR is not only involved in the generation of inducible Tregs but also enhances the suppressive capacity of existing Treg.

The possibility to differentially control Treg with anti-inflammatory properties and self-reactive conventional effector T cells by activation of NR is a potent basis to restore imbalances of immune regulation, e.g. during T cell-mediated autoimmune diseases. However, despite these promising results, NR are known to have strong metabolic properties, which limits the dosage to modulate pro- and anti-inflammatory activity. With regard to clinical use of LXR ligands, it is of interest to note, that the tolerability of pharmaceutical LXR activation is currently addressed in a clinical trial (NCT02922764) targeting LXR activation in the context of cancer treatment. These data will further reveal the potential of LXR ligands in treatment of human diseases. In addition, our findings further strengthen the therapeutic potential of LXR to ameliorate T cell-mediated chronic inflammatory diseases.

## Materials and methods

### Mice

Mice were maintained under specific pathogen–free conditions at the animal facility of the University of Münster (ZTE, Münster, Germany) or were purchased from either Charles River Laboratories (Sulzfeld, Germany) or from Harlan Laboratories (Horst, Netherlands). All animal experiments were performed according to the guidelines of the animal ethics committee and were approved by the governmental authorities of Nordrhein-Westfalen, Germany. LXRα/β^KO^ mice were generated by mating LXRα^KO^ [54] and LXRβ^KO^ [26] mice, both acquired from The Jackson Laboratory (USA).

### Nuclear receptor activation

All NR ligands were reconstituted and stored according to the supplier’s instructions. Cell culture assays were carried out applying 2μM T0901317 (Tocris), 3μM GW3965 (Tocris) or 2.5μM 22(R)-OHC (Sigma-Aldrich). Mice were orally administered 20 mg/kg body weight GW3965 (Tocris) prepared in 0.5% carboxymethylcellulose (CMC) or vehicle only (DMSO in 0.5% CMC) by daily oral gavage.

### Isolation of CD4+ T cells

Splenic CD4+ T cells were isolated by immunomagnetic separation using MACS microbeads (Miltenyi Biotec) according to the manufacturer’s instructions.

### Isolation of LN and Peyer’s Patches lymphocytes

LN and individual PPs were carefully excised, washed and grinded through a metal cell strainer. PPs were incubated for 15 min in Spleen Dissociation Medium (Miltenyi Biotech) at 37°C while shaking at 250 rpm. Both LN and PPs were subsequently grinded through a 40 μm nylon mesh cell strainer and washed twice.

### Murine Th cell differentiation

Purified CD4+ cells were stimulated with 4 μg/ml plate-bound anti-CD3 (eBioscience, Clone: 145-2C11) and 1 μg/ml soluble anti-CD28 (BD, Clone: 37.51;). For Th17 differentiation, cells were cultured in the presence of 5 ng/ml recombinant human TGFβ (R&D Systems), 20 ng/ml murine IL-6 (eBioscience), 10 μg/ml anti-IFNγ (eBioscience, Clone: XMG1.2), and 10 μg/ml anti-IL-4 (eBioscience, Clone 11B11). For Th1 differentiation, cells were cultured for up to 7 d with 10 ng/ml IL-12 (PeproTech) and 10 μg/ml anti-IL-4. For Treg induction purified mouse CD4+ T cells were stimulated with 1 ng/ml rhTGF-β for 72 h if not indicated differently. Cells were restimulated and intracellularly stained for flow cytometric analysis.

### Suppression assays

Suppression assays were performed as previously described [55]. Briefly, splenic CD4+CD25+ Treg and CD4+CD25- responder T cells (Tresp) of C57BL/6 mice were isolated by MACS (Miltenyi Biotec, Bergisch Gladbach, Germany) or by nylon wool enrichment, respectively. Purified Tresp cells were labeled with 2.5 μM Cell Proliferation Dye eFluor 670 (eBioscience, Frankfurt, Germany). Labeled Tresp cells (0.5 × 10^5^ cells) were cultured alone or together with CD4+CD25+ Treg cells (mixed at a 2:1 ratio) in the presence of anti-CD3/CD28 beads (cell to bead ratio 30:1; Dynal Biotech, Hamburg, Germany) and 1.5 μM GW3965 or vehicle control (DMSO), respectively. Flow cytometric analysis of proliferation was performed after 96 hours of coculture. Experiments were performed in triplicates. Percent suppression was calculated using the following formula: ((proliferation of T_Resp_ alone–proliferation of T_Resp_ cells cultured with Treg)/proliferation of T_Resp_ alone) x 100.

### Flow cytometry and antibodies

For intracellular staining, T cells were restimulated with 5 ng/ml PMA (Cayman Chemical) and 200 ng/ml ionomycin (Cayman Chemical) for 4 h in the presence of GolgiPlug (BD Pharmingen). Subsequently, surface staining was performed at 4°C for 30 min. Cells were fixed and permeabilized using Cytofix/Cytoperm plus Fixation/Permeabilization Kit (BD Pharmingen). For analysis of intranuclear markers, cells were fixed and stained using the FoxP3 intranuclear staining kit (eBioscience), again incubating cells at 4°C for 30 min both during fixation and intranuclear staining. Antibodies are summarized in Table 1. Analysis was performed using a Gallios Flow Cytometer (Beckman Coulter) and results were analyzed with FlowJo software (Tree Star).

**Table 1:** Antibodies used in this study.

Application	Antigen	Clone	Company
**Flow-cytometry**	HELIOS	22F6	Biolegend
	CD4	GK1.5	Biolegend
	CD25	PC61.5	eBioscience
	Foxp3	FJK-16s	eBioscience
	IFNγ	XMG1.2	eBioscience
	IL-17A	eBio17B7	eBioscience
	CTLA-4	UC10-4B9	Biolegend

### Cytokine detection

Cytokines in cell culture supernatants were detected using Enzyme-linked Immunosorbent Assay (ELISA) Ready-SET-Go!® (eBioscience) and Luminex® Screening Assay (R&D systems) according to the manufacturer’s instructions. Analysis of Luminex® assay was performed on a Bio-Plex® MAGPIX™ Multiplex Reader (Bio-Rad) according to the manufacturer’s instructions.

### Statistical analysis

All results are presented as the mean±SEM. We performed statistical analyses using Student's t-test for normally distributed data or Mann-Whitney test for non-normally distributed data sets. P < 0.05 (*) was considered statistically significant; p < 0.01 (**) and p < 0.001 (***) highly significant.

## Conclusions

We could demonstrate that LXR activation reduces effector T cell responses (Th1 and Th17) while concomitantly enhancing Treg differentiation. Importantly, LXR activation not only resulted in increased numbers of Treg but also promoted their capacity to suppress effector T cell proliferation. These data allow to speculate on the potential of therapeutically targeting LXR in T cells and Treg, respectively, to ameliorate T cell-mediated chronic inflammatory diseases.

## Supporting information

S1 Fig GW3965 reduces proinflammatory cytokine production during regulatory T cell differentiation and suppression assays. (a+b) Purified CD4+ T cells from WT mice were subjected to *in vitro* Treg-differentiation in the absence (w/o) or presence of LXR agonists GW3965 (3μM) for 72 hours (n = 6). (a) Cytokine production in the supernatant was determined after 72h by ELISA. (b) Cytokine production in the supernatant was determined after 72h by Luminex® Screening Assay. (c+d) Suppression assays were performed by coculturing murine splenic Treg (Treg; CD4+CD25+) with allogenic responder T cells (Tresp; CD4+CD25-) in a 2:1 ratio in the presence of anti-CD3/CD28 beads (cell to bead ratio = 30:1) and GW3965 (1.5 μM) or vehicle control (DMSO), respectively (n = 6). (c) Cytokine production in the supernatant was determined after 72h by ELISA. (d) Cytokine production in the supernatant was determined after 72h by Luminex® Screening Assay. Data shows pooled results of two individual experiments. Graphs show percentage ± SEM. *p<0.05 **p<0.01.
(EPS)

S1 Table ARRIVE guidelines checklist. (PDF)
